# ADMANI: Annotated Digital Mammograms and Associated Non-Image Datasets

**DOI:** 10.1148/ryai.220072

**Published:** 2022-12-21

**Authors:** Helen M. L. Frazer, Jennifer S. N. Tang, Michael S. Elliott, Katrina M. Kunicki, Brendan Hill, Ravishankar Karthik, Chun Fung Kwok, Carlos A. Peña-Solorzano, Yuanhong Chen, Chong Wang, Osamah Al-Qershi, Samantha K. Fox, Shuai Li, Enes Makalic, Tuong L. Nguyen, Daniel F. Schmidt, Prabhathi Basnayake Ralalage, Jocelyn F. Lippey, Peter Brotchie, John L. Hopper, Gustavo Carneiro, Davis J. McCarthy

**Affiliations:** From St Vincent's BreastScreen (H.M.L.F., J.S.N.T., P.B.R., J.F.L.), Department of Surgery (J.F.L.), and Department of Radiology (P.B.), St Vincent's Hospital Melbourne, 41 Victoria Parade, Fitzroy, VIC 3065, Australia; BreastScreen Victoria, Melbourne, Australia (H.M.L.F., R.K.); Bioinformatics & Cellular Genomics Unit, St Vincent's Institute of Medical Research, Fitzroy, Australia (M.S.E., K.M.K., B.H., C.F.K., C.A.P.S., D.J.M.); School of Computer Science, Australian Institute for Machine Learning, University of Adelaide, Adelaide, Australia (Y.C., C.W., G.C.); Centre for Epidemiology & Biostatistics, Melbourne School of Population and Global Health (O.A.Q., S.K.F., S.L., E.M., T.L.N., D.F.S., J.L.H.), Department of Data Science and AI, Monash University, Melbourne, Australia (D.F.S.); and Melbourne Integrative Genomics, School of Mathematics and Statistics/School of BioSciences, Faculty of Science (D.J.M.), University of Melbourne, Melbourne, Australia.

**Keywords:** Mammography, Screening, Convolutional Neural Network (CNN)

## Abstract

*Supplemental material is available for this article.*

**Keywords:** Mammography, Screening, Convolutional Neural Network (CNN)

Published under a CC BY 4.0 license.

See also the commentary by Cadrin-Chênevert in this issue.

**Summary**

The ADMANI datasets are large-scale, multicenter, clinically curated breast screening mammographic datasets created for artificial intelligence algorithm development.

**Key Points**

■ The Annotated Digital Mammograms and Associated Non-Image data (ADMANI) datasets comprise 4 411 263 images from 629 863 patients.
■ The ADMANI datasets provide strong ground truths with histopathologic proof of cancer and 2-year interval history for noncancer.
■ A subset will be available for the Radiological Society of North America Breast Cancer Detection AI Challenge.

## Introduction

Breast cancer is the most common cancer in women globally (1). The Australian national screening program offers free biennial mammographic screening targeted to women aged 50–74 years (available from age 40 years), with approximately 1 million women screened annually (2). The program has successfully led to a 41%–52% reduction in mortality for screening participants and a 21% reduction in population-level breast mortality (3).

Recent studies demonstrated that artificial intelligence (AI) may detect breast cancer on mammograms, approaching radiologist-level performance in standalone mode and improving radiologist performance in support mode (4,5). However, current evidence relies on small, retrospective, cancer-rich datasets (6). The potential for AI in the screening population is also being explored (7–9). Larger-scale, well-curated image datasets enhanced with associated demographic and clinical nonimage data and integrated with real-time deployments in clinical operations are now crucial for the future development and translation of AI algorithms into clinical practice. Globally, there are only a few mammographic datasets available for such research, as outlined in Table S1.

This article describes the curation of annotated digital mammogram and associated nonimage datasets (ADMANI1, ADMANI2, and ADMANI3) containing 4 411 263 images from 629 863 women and 1 048 345 screening episodes performed at the state screening service. These datasets were developed by the Transforming Breast Cancer Screening with AI (BRAIx) program to enable the development of AI-based algorithms to aid breast cancer detection in the mammographic screening population and support risk-based screening (10). We intend to continue growing the datasets over subsequent years.

## Materials and Methods

### Ethics

Use of the ADMANI datasets is governed under the executed BRAIx Multi-Institutional Agreement, with approvals by the human research ethics committee (approval nos. LNR/18/SVHM/162 and LNR/19/SVHM/123). All women sign a consent form at screening registration that provides for the use of the de-identified data for research purposes. A unique identifier is used for the purposes of the ADMANI datasets, with all image and nonimage data de-identified.

### Screening Episode Structure

**Figure 1:**
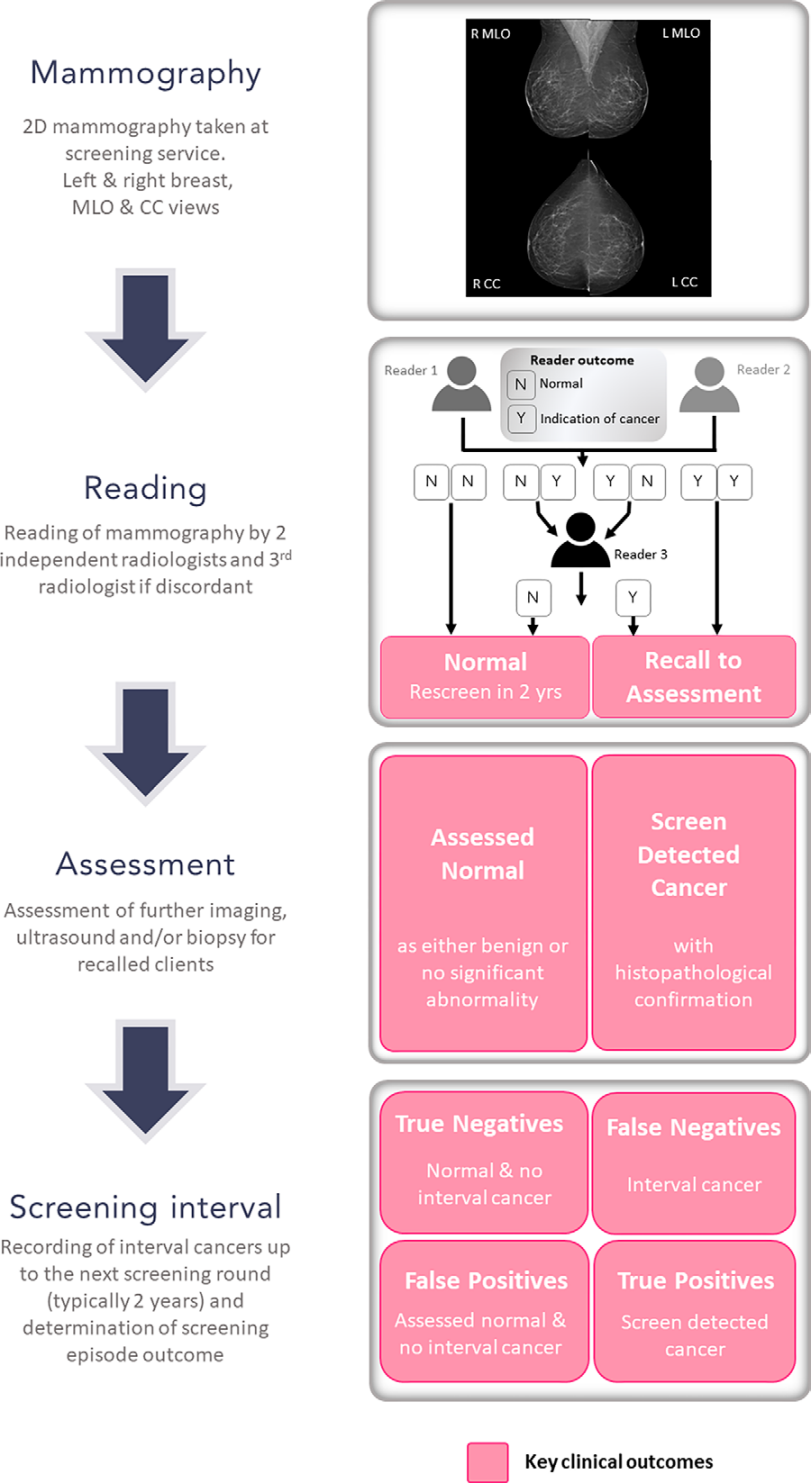
Overview of a BreastScreen Victoria screening episode with key clinical outcomes. CC = craniocaudal, L = left, MLO = mediolateral oblique, R = right, 2D = two-dimensional.

The datasets are structured around an individual screening episode of a woman attending BreastScreen Victoria. A screening episode is defined as a single screening round that includes mammography, reading, assessment, and the subsequent 2-year screening interval (Fig 1).

### Details for Each Step Are Below

***Mammography.—***Screening episodes with a minimum of four standard full-field digital mammograms comprising one mediolateral oblique and one craniocaudal image of each breast were included, including those with implants, prior surgery, and prior cancers. Screening episodes were excluded if the outcome could not be determined or if matching images and nonimage data were lacking. For screening episodes with multiple imaging attempts, the final was selected.

***Reading.—***Each set of mammograms was read independently by two breast imaging radiologists who registered either an indication for cancer with image annotation or an all clear.

If both radiologists registered an indication, the woman was recalled for assessment; if both registered all clear, the woman was recommended for routine rescreening (typically in 2 years). When both radiologists disagreed, a third radiologist aware of the discordance read the set of mammograms to make the final decision.

***Assessment.—***Women recalled for assessment underwent further imaging, including digital breast tomosynthesis and/or US, and a needle biopsy if required, to determine cancer diagnosis. A very small number of women required open diagnostic biopsy to complete assessment.

***Screening interval.—***Interval cancer was determined if women recommended for routine rescreening at the reading or assessment stages were diagnosed with breast cancer during the 2-year interval prior to their next screening episode.

### Ground Truth

Ground truth for cancer was based on histopathologic findings, predominantly from both biopsy assessment and subsequent surgery for confirmation, or the reporting of interval cancer during the 2-year screening interval.

Ground truth for noncancers was an all clear outcome after reading or assessment and the subsequent screening interval.

### Key Clinical Variables

For each screening episode, data were examined at four levels: individual reader outcomes, consensus reading outcome, assessment outcome, and final screening episode outcome. Individual reader outcomes were recorded for each episode as follows: *(a)* all clear: a reader records no indication for cancer on left and right breast images; and *(b)* indication for cancer: a reader records an indication for cancer on left, right, or both breast images (in event of bilateral cancer indication). Consensus reading outcomes were assigned for each episode as follows: *(a)* normal: episodes assigned as all clear by both readers, or by the third reader for discordant opinions; and *(b)* recall for assessment: episodes in which both readers, or the third reader for discordant opinions, recorded indications for cancer.

For women recalled, the episode was assigned the most prognostically significant assessment outcome, as follows: *(a)* assessed normal: an episode where the woman was recalled but findings were assessed as either benign or having no significant abnormality; *(b)* screen-detected cancer: an episode where the woman was recalled and assessed as having ductal carcinoma in situ and/or an invasive malignancy, with histopathologic confirmation following surgery.

The final screening episode outcome was recorded based on the reading and assessment outcomes and the woman's history in the subsequent 2-year interval between screening rounds, as follows: *(a)* false negative: an episode assigned normal following consensus reading or assessment (benign or no significant abnormality), but a cancer registry notification indicates the woman developed breast cancer in the subsequent 2-year interval between screening episodes (ie, interval cancer); *(b)* true negative: an episode in which the reading outcome is normal, and the woman was not diagnosed with an interval cancer; *(c)* false positive: an episode in which the reading outcome is a recall for assessment, the assessment outcome is normal, and the woman was not diagnosed with an interval cancer; *(d)* true positive: an episode in which the reading outcome is recall for assessment and the assessment outcome is a (biopsy-proven) screen-detected cancer.

**Figure 2:**
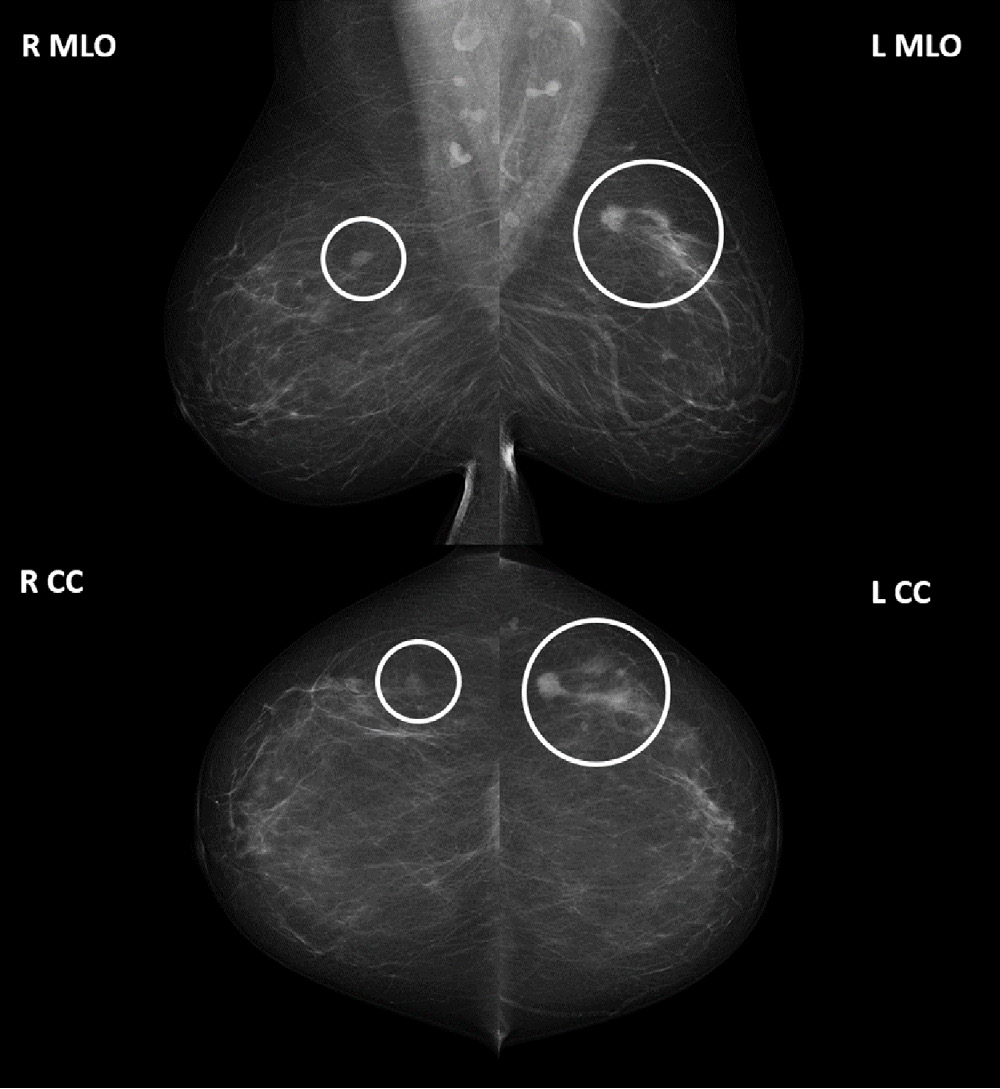
A typical four-view mammogram from a screening episode. The final screening episode outcome for this woman was true positive, with a screen-detected cancer on the left breast as annotated. The left breast images were classified with this final screening episode outcome along with the associated reading and assessment outcomes. The lesion on the right breast was also indicated for assessment and determined to be benign. As such, the right breast images, although associated with a true-positive screening episode, were classified with the less prognostically significant reading (recall for assessment) and assessment (assessed normal with benign lesion) outcome. CC = craniocaudal, L = left, MLO = mediolateral, R = right.

The images of the breast associated with the final screening episode outcome were classified accordingly along with the associated reading and assessment outcomes. The images of the other breast, while associated with the final screening episode outcome for the woman, were also classified (except in the occurrence of a bilateral cancer) with the less prognostically significant reading and assessment outcomes (Fig 2).

## Resulting Dataset

### Dataset Characteristics

The ADMANI datasets, comprising three subsets (titled as ADMANI1, ADMANI2, and ADMANI3), contain more than 4.4 million images (629 863 women) from more than 1 million screening episodes, including both image and associated nonimage data. There are a total of 22 270 screen-detected cancer images (10 247 screening episodes) and 6641 interval cancer images (3097 screening episodes) in the datasets. Of the 10 247 screening episodes with a screen-detected cancer, 8128 of 10 247 (79%) are invasive and 2119 of 10 247 (21%) are ductal carcinoma in situ. Table S2 describes the characteristics of the cancers in the dataset.

ADMANI1 was established as a cancer-rich dataset for early in silico model development and testing. It consists of 228 901 images from 54 251 episodes.

ADMANI2 and ADMANI3 were established as large-scale, population-based, longitudinal resources that reflect the real-world screened population with low incidence of breast cancer and include diagnosed interval cancers. The ADMANI2 dataset consists of 2 095 085 images from 497 440 episodes from women screened in the state during 2016 and 2017. The ADMANI3 dataset consists of 2 087 277 images from 496 654 episodes from women screened in the state during 2018 and 2019. These datasets enable real-world evaluation of AI models and their application at different operating points throughout the screening episode.

The number of episodes by final screening episode outcome and age group in these datasets is outlined in Tables 1 and 2, respectively.

**Table 1:**
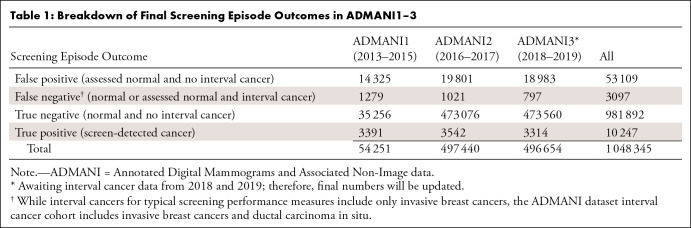
Breakdown of Final Screening Episode Outcomes in ADMANI1–3

**Table 2:**
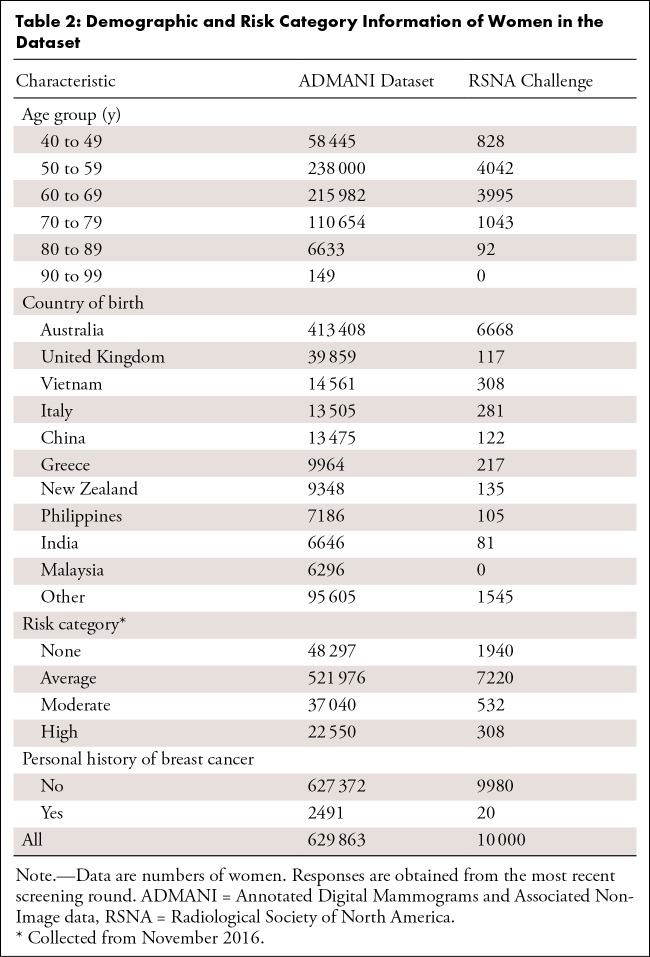
Demographic and Risk Category Information of Women in the Dataset

### Image Data

**Figure 3:**
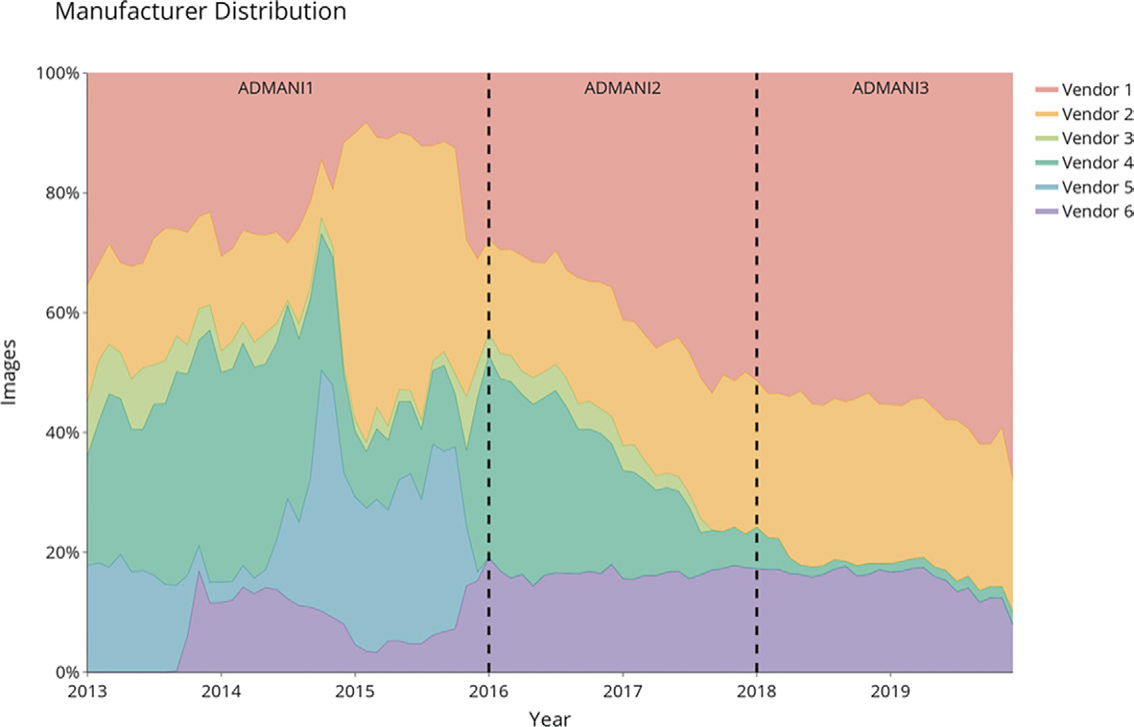
Manufacturer distribution across the datasets. ADMANI = Annotated Digital Mammograms and Associated Non-Image data.

The ADMANI datasets include “for presentation” digital screening mammograms acquired from six different manufacturers: Siemens, Hologic, Philips, Fujifilm, Sectra Imtec, and Konica Minolta (Fig 3). Imaging data include kilovoltage peak, x-ray tube current in milliamperes, and exposure time in milliseconds. Expert radiologist annotations are provided where available from the original read of the mammogram (drawn region of interest or localization by quadrant).

### Nonimage Data

The ADMANI datasets include patient, radiologist reading, and histopathologic data. Patient data collected at the time of the episode include age, country of birth, risk category (average, moderate, and high), symptoms (eg, lump, nipple discharge), and other personal characteristics, such as the use of hormone replacement therapy. Radiologist reading data include the lesion side and grade. Histopathologic data contain surgical specimen results, including lesion subtype.

## Discussion

The development of large-scale, longitudinal datasets is essential to facilitating the translation of AI into clinical practice. These datasets require clinical input and structured processes to ensure appropriate links between image and nonimage data. As AI methods advance and more questions are posed, there will be considerable value in well-curated, real-world datasets such as the ADMANI datasets.

The ADMANI datasets are currently supporting real-world retrospective and prospective studies, providing the flexibility to evaluate feasibility of AI deployment within different stages of the screening pathway. Potential applications for AI in mammographic screening include triaging, replacing certain radiologist readers, or decision support. Each potential application requires thorough evaluation of the clinical and economic impact in a population setting. The ADMANI datasets offer an avenue for such evaluation.

There remain a number of limitations to dataset curation for AI development. More research is required to understand the nature of interval cancers that could be detected with AI versus those that arise de novo in the screening interval. Additionally, the availability of digital breast tomosynthesis images and breast US images are yet to be included.

The nontransformed image and nonimage data that established the ADMANI datasets are being used by the ADMANI program under license agreement with the state screening program. We are currently developing the necessary funding and governance to support future availability of these datasets. A subset of 40 000 images from 10 000 episodes will be provided for the Radiological Society of North America Mammography Breast Cancer Detection AI Challenge, launching on November 28, 2022. The challenge training dataset will be made public when the challenge is launched and will remain available to researchers when the challenge concludes. The 10 000 episodes will be randomly selected from the dataset from a 3-year period.
